# Alumni Association, University of Buffalo — Department of Medicine

**Published:** 1903-07

**Authors:** Thomas H. McKee


### Alumni Association, University of Buffalo — Department of Medicine.

REPORTED BY THOMAS H. McKEE, M. D., Secretary.

The twenty-eighth annual meeting of the Alumni Association of the University of Buffalo was called to order in Alumni Hall by the president, A. W. Bayliss, at 2.30 p.m., May 5, 1903.

After the reading of the minutes of the previous session, and receiving reports of officers and various committees, the meeting proceeded with the election of officers for the ensuing year with the following result: president, A. W. Henchell, '89, Rochester; 1st vice-president, Fridolin Thoma, '87, Buffalo; 2d vice-president, Henry T. Benham, '90, Honeoye Falls; 3d vice-president, Jane W. Carroll, '91, Buffalo; 4th vice-president, George W. Himmelsbach, '91, Buffalo; 5th vice-president, C. J. Reynolds, '90, Buffalo. Board of Trustees—D. A. Currie, '64, Englewood, N. J.; A. W. Bayliss, '89, Buffalo; William Warren Potter, '59, Buffalo; D. W. Harrington, '71, Buffalo; F. H. Moyer, '72, Moscow. Representative on the Council—Ernest Wende. Executive Committee—Albert T. Lytle, '93, Chairman, Buffalo; Grover W. Wende, '89, Buffalo; Franklin W. Barrows, '93, Buffalo; A. W. Henchell, *ex-officio*, Rochester; John Parmenter, *ex-officio*, Buffalo.

By request of Dr. Stoddard, who was unavoidably absent, Dr. Mann read a memorial of Dr. William H. Mason, formerly professor of physiology in the university.

IN MEMORIAM—WILLIAM H. MASON.

On June 1, 1902, passed away at Vergennes, Vt., Professor William H. Mason, who for many years filled the chair of physiology in this institution.

Professor Mason was born in New England, of Puritan ancestry, and his early life was spent, and his literary and classical education pursued, amid the traditions of that sturdy section. Endowed with a fondness for the natural sciences and for physical research, he adopted the profession of medicine as his life work. His medical studies were terminated at the medical department of the University of Buffalo, where he received the degree of Doctor of Medicine in 1859.

On the completion of his medical course, he settled for a short time in Buffalo, N. Y., whence he went, subsequently, in company with Dr. Austin Flint, Jr., to Europe, for the purpose of pursuing further the study of physiology. He was, for a considerable time, a pupil of M. Claude Bernard. Returning from this period of foreign study and observation, he settled in Norwich, Conn., where he subsequently married Eliza Whittaker, daughter of the late Horace Whittaker, of "Sunnyside." In 1861, he accepted the position of professor of physiology in the University of Buffalo, which chair he filled most acceptably during the period of twenty-five years, finally resigning in 1886.

As a teacher, Professor Mason was earnest and forceful, and believed in and pursued physical demonstrations as essential to the comprehension of complicated physiological deductions. He was of a retiring disposition. This characteristic led him to decline any position which would bring him into prominence. Endowed with native ability and with quick perceptions, his fondness for study kept him apace with the most progressive thought of his time. Of domestic tastes and inclinations, his devotion to his home was marked, and to the members of his family, and to those whom he admitted to a personal friendship, he was ever loyal and devoted.

The last ten years of his life were saddened by the death, successively, of his daughter, wife and son. The breaking up of his home and of the ties, which had so long held him to it as the center around which the events of his daily life had clustered, rudely disturbed the current of a quiet life and made each succeeding year bear more and more heavily upon him, until he sunk beneath the increasing weight of the burden and passed quietly away.

This brief memorial is the earnest tribute of one who, for seventeen years, was associated with Professor Mason as a colleague, and who enjoyed his confidence and friendship. The sadness and regret which we feel in the passing away of our friend, is deepened by the thought that his last years were darkened by the sombre shadows of great sorrow, and passed in seclusion far from the scenes of the happiest portion of his life.

Professor William T. Sedgwick, Ph.D., of the Massachusetts Institute of Technology, then presented very comprehensively the subject,

PROTECTION OF PUBLIC HEALTH BY FILTRATION OF MUNICIPAL WATER SUPPLIES.

The paper was discussed by Drs. Walter D. Greene, commissioner of health, H. M. Hill, city chemist, H. U. Williams, professor of bacteriology, University of Buffalo, Dr. Campbell, of Niagara Falls, and Dr. Fell, of Buffalo.

On motion the meeting adjourned sine die.

